# Ubiquitin-proteasome dependent degradation of GABA_A_α1 in autism spectrum disorder

**DOI:** 10.1186/2040-2392-5-45

**Published:** 2014-09-01

**Authors:** Amanda Crider, Chirayu D Pandya, Diya Peter, Anthony O Ahmed, Anilkumar Pillai

**Affiliations:** 1 Department of Psychiatry and Health Behavior, Medical College of Georgia, Georgia Regents University, 997 St. Sebastian Way, Augusta, GA 30912, USA; 2 Department of Psychiatry, Weill Cornell Medical College, 21 Bloomingdale Rd, White Plains, NY 10605, USA

**Keywords:** Autism, GABA_A_ receptor, Ubiquitination, SYVN1, Neurons

## Abstract

**Background:**

Although the neurobiological basis of autism spectrum disorder (ASD) is not fully understood, recent studies have indicated the potential role of GABA_A_ receptors in the pathophysiology of ASD. GABA_A_ receptors play a crucial role in various neurodevelopmental processes and adult neuroplasticity. However, the mechanism(s) of regulation of GABA_A_ receptors in ASD remains poorly understood.

**Methods:**

Postmortem middle frontal gyrus tissues (13 ASD and 13 control subjects) were used. *In vitro* studies were performed in primary cortical neurons at days *in vitro* (DIV) 14. The protein levels were examined by western blotting. Immunofluorescence studies were employed for cellular localization. The gene expression was determined by RT-PCR array and qRT-PCR.

**Results:**

A significant decrease in GABA_A_α1 protein, but not mRNA levels was found in the middle frontal gyrus of ASD subjects indicating a post-translational regulation of GABA_A_ receptors in ASD. At the cellular level, treatment with proteasomal inhibitor, MG132, or lactacystin significantly increased GABA_A_α1 protein levels and Lys48-linked polyubiquitination of GABA_A_α1, but reduced proteasome activity in mouse primary cortical neurons (DIV 14 from E16 embryos). Moreover, treatment with betulinic acid, a proteasome activator significantly decreased GABA_A_α1 protein levels in cortical neurons indicating the role of polyubiquitination of GABA_A_α1 proteins with their subsequent proteasomal degradation in cortical neurons. Ubiquitination specific RT-PCR array followed by western blot analysis revealed a significant increase in SYVN1, an endoplasmic reticulum (ER)-associated degradation (ERAD) E3 ubiquitin ligase in the middle frontal gyrus of ASD subjects. In addition, the inhibition of proteasomal activity by MG132 increased the expression of GABA_A_α1 in the ER. The siRNA knockdown of SYVN1 significantly increased GABA_A_α1 protein levels in cortical neurons. Moreover, reduced association between SYVN1 and GABA_A_α1 was found in the middle frontal gyrus of ASD subjects.

**Conclusions:**

SYVN1 plays a critical role as an E3 ligase in the ubiquitin proteasome system (UPS)-mediated GABA_A_α1 degradation. Thus, inhibition of the ubiquitin-proteasome-mediated GABA_A_α1 degradation may be an important mechanism for preventing GABA_A_α1 turnover to maintain GABA_A_α1 levels and GABA signaling in ASD.

## Background

Autism spectrum disorder (ASD) is among the most devastating neurodevelopmental disorders with a prevalence of about 1% of population worldwide. Although many theories have been suggested to explain the neurobiology of ASD, the exact mechanism is still not understood. Recent studies indicate the role of the inhibitory neuronal circuit dysfunction in the pathophysiology of ASD [1]. γ-amino butyric acid (GABA) is the major inhibitory neurotransmitter in the brain, and studies have shown an association between ASD and GABAergic system [2]. GABA_A_ receptors mediate the fast inhibitory neurotransmission within the CNS, and most GABA_A_ receptors are composed of 2α, 2β, and 1γ or δ-subunit [3]. GABA_A_α1 is expressed ubiquitously in the brain [4] and is present over 60% of cortical GABA_A_ receptors [5]. It is considered to be responsible for sedative effects of positive allosteric modulators of the GABA_A_ system, such as diazepam [6]. Since GABA_A_α1 is the major receptor in the GABA system, its dysfunction significantly affects GABA signaling and therefore, brain physiology [7-10]. A significant reduction in GABA_A_α1 protein levels has been found in the frontal cortex of ASD subjects [11]. However, the mechanism(s) of GABA_A_α1 regulation in ASD is still not clear.

The ubiquitin-proteasome system (UPS) is a major non-lysosomal proteolytic process that regulates the levels of cellular proteins including those involved in neuronal growth and function [12]. Moreover, UPS has been shown to regulate a number of GABA receptors suggesting a possible relationship between UPS and GABAergic system [13-15]. The UPS consists of concerted actions of three classes of enzymes that link the polypeptide co-factor, ubiquitin (Ub) onto proteins to mark them for degradation [16,17]. In the first step, the C-terminus of Ub forms a thioester bond with the catalytic cysteine of an E1 Ub-activating enzyme. In the second step, Ub is transferred from the E1 to the catalytic cysteine of the E2, Ub-conjugating enzyme. Finally, the E2 - Ub conjugate cooperates with an E3 (ligase) to transfer Ub to the substrate. Moreover, the interaction between an E3 ligase and its target molecule is a key step in determining the selectivity of UPS for a target molecule and its proteasomal degradation.

The present study investigated the role of ubiquitination in the regulation of GABA_A_α1 in ASD. We have examined the hypothesis that GABA_A_α1 protein levels are degraded through a UPS-mediated pathway in ASD. We tested the above hypothesis in postmortem middle frontal gyrus samples from ASD and control subjects. The middle frontal gyrus contains the core portion of dorsolateral prefrontal cortex, a region primarily associated with cognition and executive functions. A large body of evidence including reports from neurocognitive as well as neuroimaging studies has implicated middle frontal gyrus in the pathophysiology of ASD [18-20].

## Methods

### Ethics statement

The Georgia Regents University (GRU) Institutional Review Board has deemed this study exempt from the full review due to the use of de-identified human postmortem brain samples, with no possibility to track back the identity of the donors. Animal use procedures were performed after being reviewed and approved by GRU, Committee on Animal Use for Research (protocol# 2010-0056). Procedures were consistent with the Association for Assessment and Accreditation of Laboratory Animal Care (AAALAC) guidelines as per Public Health Service Policy on Humane Care and Use of Laboratory Animals.

### Postmortem samples

Postmortem brain tissues from middle frontal gyrus of ASD (N = 13) and control (N = 13) subjects were received from the NICHD Brain and Tissue Bank for Developmental Disorders at the University of Maryland, Baltimore, MD, USA. Detailed description on the demographics of samples is given in Additional file 1: Table S1. None of the controls had any known history of neuropsychiatric disorders or illicit drug use. 9 out of 13 subjects with ASD had information on Autism Diagnostic Interview-Revised (ADI-R). Confounding variables such as PMI, refrigeration interval, age at death, RNA integrity, and brain pH did not show any significant difference between ASD and control subjects (Table 1). The brain samples were shipped frozen and stored at -80°C until analysis. Brain tissue was homogenized in a tissue lysis buffer containing 50 mM Tris-HCl (pH 7.5), 2 mM EDTA, 150 mM NaCl, 1.0% Triton X-100, 1.0% sodium deoxycholate, 0.1% sodium dodecyl sulfate (SDS), 6 μM PMSF, and protease inhibitor cocktail (Sigma) followed by centrifugation at 13,000 rpm for 10 min at 4°C. The supernatant was used for protein estimation by the bicinchoninic acid method (BCA Protein Assay Kit, Thermo).

**Table 1:** Demographic characteristics of postmortem brain samples

**Variable**	**Control**	**ASD**
Age (years)	11.70 ± 1.584	11.80 ± 1.609
PMI (h)	14.46 ± 2.171	19.00 ± 2.776
Gender (F/M)	1/12	0/13
Storage (days)	4,287 ± 638.7	2,829 ± 397.7
RIN	5.55 ± 0.71	6.84 ± 0.53
pH	5.95 ± 0.06	6.11 ± 0.07

### Primary cortical neurons

Timed pregnant CD-1 mice were purchased from Charles River Laboratories (Wilmington, MA, USA). Cerebral cortical neurons were prepared as described previously [21]. Briefly, cerebral cortices from embryos at E16 were aseptically dissected and plated at 3.5 × 10^5^ cells per well on polyethyleneimine-coated 6-well plates. Neurons were cultured in Neurobasal medium supplemented with 2 mM L-glutamine, B27 and antibiotics (Invitrogen). The media was replaced with Neurobasal supplemented with B27 minus antioxidants, glutamine, and antibiotics on the third day *in vitro* (DIV 3). Treatment of neurons was conducted at DIV 14. The following pharmacological treatments were used: MG132 (20 μM); lactacystin (50 μM); or betulinic acid (20 μg/mL) (all the reagents were from Tocris Biosciences, Minneapolis, MN, USA). At the end of the treatments, cells were washed in Phosphate Buffered Saline (PBS) and lysed in ice-cold lysis buffer supplemented with protease inhibitor cocktail (Sigma) for immunoblotting.

### Immunoblotting

Protein samples (30 to 40 μg) were subjected to SDS-PAGE and transferred onto a nitrocellulose membrane. The membrane was then blocked for 1 h in PBS with the detergent Tween 20 and 5% non-fat milk or 5% BSA followed by overnight incubation with a primary antibody. The primary antibodies used were: anti-GABA_A_α1 (1:1,000, PhosphoSolutions, Aurora, CO, USA); anti-GABA_A_γ2 (1:200; Santa Cruz Biotechnology); anti-GABA_A_α2 (1:200; Santa Cruz Biotechnology); anti-GABA_A_α3 (1:200; Santa Cruz Biotechnology); anti-SYVN1 (1:1,000, Sigma) or anti- ubiquitin Lys48-specific (clone Apu2, 1:1,000, Millipore). Following washing, the membranes were incubated with secondary antibody for 1 h. We used enhanced chemiluminescence detection reagent kit (Thermo Scientific) to detect the proteins. The intensity of the bands was quantified using densitometry software (Image J, NIH). The immunoblot data was corrected for corresponding GAPDH (1:5,000, Cell Signaling) values. Immunoprecipitation was performed using the Crosslink Immunoprecipitation Kit (Pierce, cat# 26147). Briefly, precipitating antibody (10 μg) was bound to protein A/G plus agarose for 1 h followed by cross linking using disuccinimidyl suberate (DDS) crosslinker. The antibody-crosslinked resin was incubated overnight with the pre-cleared lysate (200 μg). Lysate preclearance was carried out using control agarose resin for 1 h. Antigen co-precipitated with antibody-crosslinked resin was eluted and subjected for SDS-PAGE analysis as described above.

### Quantitative reverse transcriptase PCR (qRT-PCR)

Total RNA was isolated from postmortem brain tissues using a commercially available kit (SV RNA Isolation, Promega, Madison, WI, USA). qRT-PCR was performed on a MasterCycler (Eppendorf) using a SuperScript III Platinum SYBR Green One-Step qRT-PCR kit (Invitrogen, Carlsbad, CA, USA). A typical reaction of a total volume of 25 μL consisted of 0.5 μL Superscript III RT/Platinum Taq mix, 12.5 μL 2X SYBR Green Reaction Mix (includes 0.4 mM of each dNTP and 6 mM MgSO4), 12.5 pMol of each of forward or reverse primers, and 4 μL DEPC-treated water, and 3ul of purified RNA. PCR amplification was done with an initial incubation at 55°C for 1,200 s, then at 95°C for 120 s followed by 35 cycles of 95°C for 15 s, 50°C for 30 s, 72°C for 30 s, and final melting curve from 55°C to 95°C with 0.2 C/s. Primer specificity was confirmed by melting curve analysis and electrophoresis of PCR products on a 2% agarose gel to confirm the presence of a single band of the predicted size. The mRNA for GABA_A_α1 and SYVN1 were normalized to two control genes (glyceraldehyde 3-phosphate dehydrogenase (GAPDH), and *ß*-actin) and a geometric mean of these genes. Primers utilized were as follows: GABA_A_α1 (forward, 5’-GGATTGGGAGAGCGTGTAACC and reverse, 5’- TGAAACGGGTCCGAAACTG), SYVN1 (forward, 5’-GTTTACAGGCTTCATCAAGG and reverse, 5’-CATGATGGCATCTGTCACAG), GAPDH (forward,5’-GAGTCAACGGATTTGGTCGT-3’ and reverse, 5’-TTGATTTTGGAGGGATCTCG-3’), and *ß*-actin (forward, 5’-GGACTTCGAGCAAGAGATGG–3’ and reverse, 5’-AGCACTGTGTTGGCGTACAG-3’).

### Small interfering RNA (siRNA)

The control as well as SYVN1 siRNA were purchased from Dharmacon Research Inc. Transfection of both siRNAs (50 nM) was performed using Effectene Transfection Reagent (Qiagen). Lysates were collected at 48 h after the transfection.

### Human Ubiquitination Pathway PCR Array

The human Ubiquitination Pathway RT^2^ Profiler PCR Array (SA Biosciences, Qiagen, Valencia, CA, USA) was used to determine the profile of genes involved in UPS pathway as per the manufacturer’s instructions. The array determines the gene expression of 84 molecules in the family of ubiquitin-activating enzymes (E1), ubiquitin-conjugating enzymes (E2), and ubiquitin ligases (E3). The integrated web-based software package was used for data analysis (http://www.SABiosciences.com/pcrarraydataanalysis.php).

### Proteasome activity assay

The proteasome activity was measured using the 20S proteasome activity assay kit (Millipore) according to the manufacturer’s instructions. Primary cortical neurons (DIV 14) were treated with vehicle (DMSO), MG132 (20 μM), or lactacystin (50 μM) for 9 h. Cells were washed with PBS and lysed in cell lysis buffer (50 mm Tris, pH 7.4, 2 mm dithiothreitol, 5 mm MgCl_2_, 2 mm ATP). Following homogenization and centrifugation, 100 μg of protein of each sample was diluted to a final volume of 100 μL with assay buffer (250 mM HEPES, pH 7.5, 5 mM EDTA, 0.5% NP-40, and 0.01% SDS) and proteasome substrate, SucLLVY-7-amido-4-methylcoumarin. The assay was based on detection of the fluorophore 7-amino-4-methylcoumarin (AMC) after cleavage from the labeled substrate. The free AMC fluorescence was quantified using a Synergy HT Multi-detection Microplate Reader (Bio-Tek Instruments, Inc., Winooski, VT, USA) at 380/460 nm and 37°C.

### Immunofluorescence

Primary cortical neurons (DIV 14) were treated with vehicle (DMSO) or MG132 (20 μM) for 9 h. Cells were washed with PBS and fixed with 4% paraformaldehyde in PBS for 30 min at room temperature. After washing with PBS, the cells were blocked with 10% goat serum in 0.2% Triton X-100/PBS for 2 h at 37°C and incubated with rabbit anti- GABA_A_α1 (1:200) and mouse anti-PDI (1:1,000; Enzo Life Sciences, Farmingdale, NY, USA) overnight at 4°C. After washing and incubation with Cy3 or Cy2-based secondary antibodies (1:200), the cells were washed extensively with PBS, and mounted with ProLong Gold Antifade Reagent with DAPI (Invitrogen). Confocal images were taken with a Zeiss LSM-510 confocal microscope. Colocalization of proteins was confirmed by z stack analysis. Data show representative single plane images.

### Statistical analyses

One-way ANOVA with Bonferroni’s multiple comparison test for post-hoc analysis or Student’s t test was used in cell culture studies. In postmortem data analysis, univariate general linear models were used to examine the differences in mRNA and protein expression levels between subjects with ASD and the controls (that is, affection status). To examine the unique effects of affection status on mRNA and protein expression, age, postmortem interval, storage time, sample pH, and/or RNA integrity number were evaluated and added to the model as potential covariates. The small sample size used to conduct the Analysis of Covariance (ANCOVA) implied a decreased power to detect effects. In order to maximize the observed power, only covariates that had at least small-to-moderate associations with the dependent variable were included in the model. Candidate covariates that were significantly correlated with mRNA or protein expression were included in the model. Partial eta-squared coefficients were computed as estimates of effect size. Exact probability (*P*) values of less than 5% were considered significant. Depending on distributional characteristics of the data, simple or non-parametric correlations were computed to examine the association of covariates and clinical variables with mRNA and protein expression levels. Pearson’s product moment correlation was used for the correlation analysis. All analyses were performed using SPSS Statistics 20 software (IBM).

## Results

### Decrease in GABA_A_α1 protein, but not mRNA levels in the middle frontal gyrus of ASD subjects

Since the postmortem findings can be significantly affected by confounding variables such as age, sex, postmortem interval (PMI), pH, and RNA integrity number (RIN), all statistical analysis was adjusted for these covariates. For each protein (or gene) expression level, insignificant covariates were removed and then a statistical model was established to correct for any significant covariates. Additional file 2: Table S2 summarizes the distributional characteristics of study variables. For each molecule, candidate covariates included in the model had a correlation magnitude of 0.10 and above.

**Figure 1:**
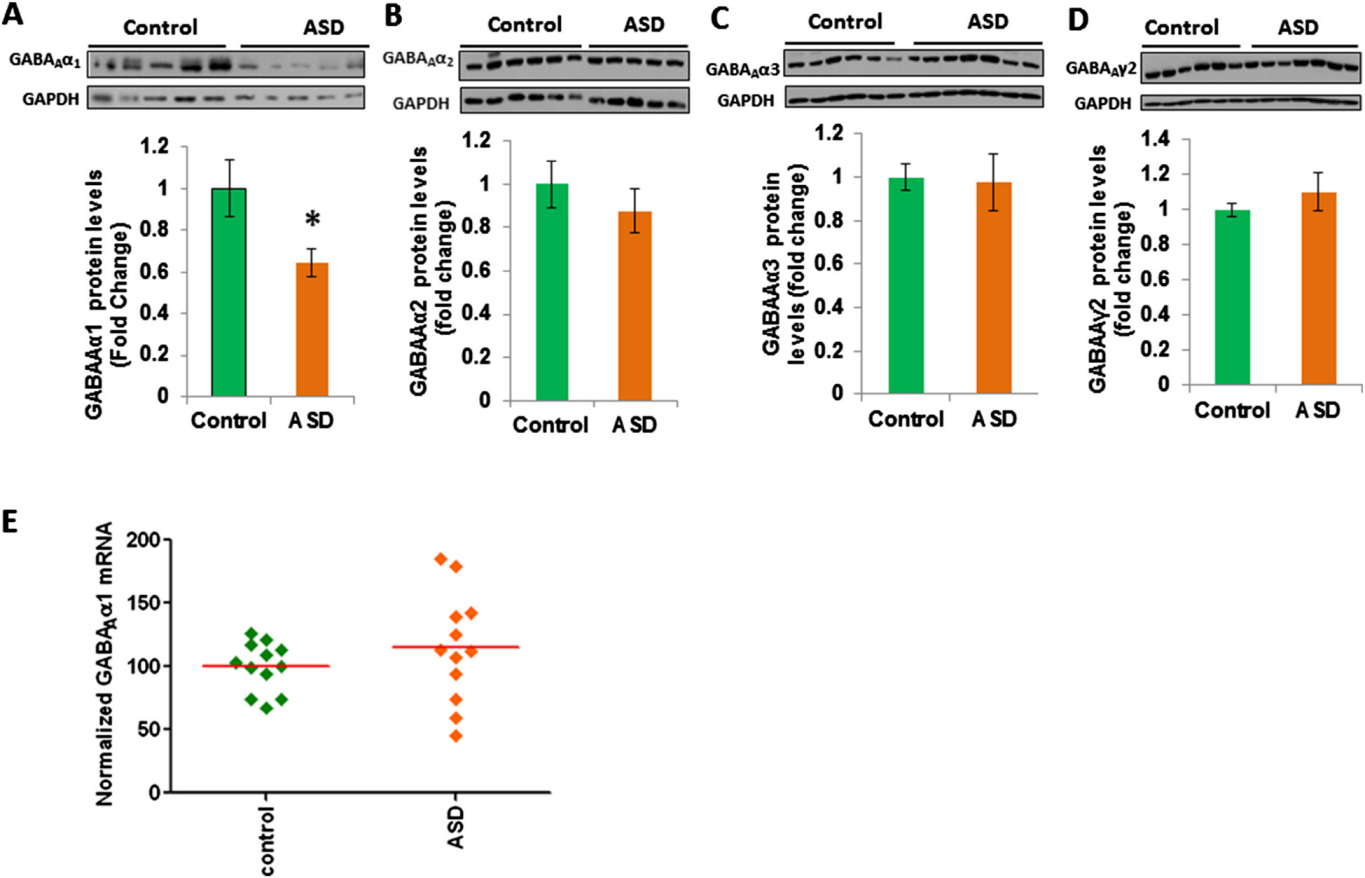
**Decreased GABA**_**A**_**α1 protein, but not mRNA expression in the middle frontal cortex of ASD subjects. (A)** GABA_A_α1 protein levels were determined by western blot analysis. The upper panel shows representative autoradiogram of GABA_A_α1 or GAPDH, and the lower panels represent fold change in normalized GABA_A_α1 protein levels. Results are mean ± SEM. *P <0.05 *vs.* controls. **(B-D)** Representative autoradiogram and fold change in protein levels of **(B)** GABA_A_α2, **(C)** GABA_A_α3, and **(D)** GABA_A_γ2 in ASD and control subjects. **(E)** mRNA was determined by qRT-PCR, and the values were normalized to the geometric mean of two control genes (glyceraldehyde 3-phosphate dehydrogenase (GAPDH), and *ß*-actin).

Expression of GABA_A_α1 was examined in the middle frontal gyrus of ASD and control subjects by western blotting (Figure 1A). Including sample pH as a covariate in the model, the predicted main effect of affection status was statistically significant (*F*(1, 21) = 4.57, *P* = 0.045, η^2^_p_ = 0.179). In contrast, the main effect of sample pH was not statistically significant (*F*(1, 21) = 0.19, *P* = 0.89, η^2^_p_ = 0.001). Subjects with ASD demonstrated lower levels of GABA_A_α1 protein expression (EMM (estimated marginal means) = 0.649, SE (standard error) = 0.113) than controls (EMM = 0.997, SE = 0.113). However, we did not find any significant change in the protein levels of GABA_A_α2, GABA_A_α3, and GABA_A_γ2 in ASD subjects as compared to controls (Figure 1B to D). To determine whether GABA_A_α1 downregulation occurred at the mRNA level, we examined the GABA_A_α1 mRNA expression in the middle frontal gyrus of ASD and control subjects (Figure 1E). An ANCOVA (between-subjects factor: affection status (ASD, control); covariates: age, storage interval, RNA integrity number) demonstrated no main effect of affection status, *F*(1, 19) = 0.056, *P* = 0.82, η^2^_p_ = 0.003. The main effects of age (*F*(1, 19) = 4.26, *P* = 0.053, η^2^_p_ = 0.183), storage interval (*F*(1, 19) = 2.61, *P* = 0.123, η^2^_p_ = 0.12), and RNA integrity number (*F*(1, 19) = 1.25, *P* = 0.277, η^2^_p_ = 0.062) were not statistically significant. No significant correlation was found between the mRNA or protein expression of GABA_A_α1 and the confounding variables, such as age at death, PMI, refrigeration interval, brain pH, or RNA integrity (Table 2). In addition, we did not observe any association between the ASD diagnostic scores and GABA_A_α1 mRNA or protein in ASD subjects. These results indicate that GABA_A_α1 levels are lower in the middle frontal gyrus of ASD subjects, which occurred at the post-translational level.

**Table 2:** **Correlations of GABA**_**A**_**α1 mRNA and protein levels with confounding variables**

	**GABA**_**A**_**α1 mRNA**				**GABA**_**A**_**α1 protein**			
	**CON**		**ASD**		**CON**		**ASD**	
	**r**	***P***	**r**	***P***	**r**	***P***	**r**	***P***
Age	-0.260	0.414	-0.329	0.296	-0.960	0.542	0.044	0.891
PMI	0.014	0.967	-0.071	0.826	-0.110	0.734	0.148	0.647
Storage	-0.456	0.136	-0.162	0.614	-0.229	0.474	-0.126	0.695
RIN	0.202	0.528	0.109	0.736	-0.190	0.553	0.451	0.125
pH	0.236	0.460	-0.312	0.324	-0.349	0.266	0.445	0.148
Social interaction			0.253	0.546			0.407	0.316
Verbal communication			0.496	0.395			-0.643	0.169
Non-verbal communication			-0.067	0.887			-0.625	0.134
Stereotyped behavior			-0.595	0.119			0.598	0.117
Abnormality of development			-0.081	0.848			0.294	0.522

Pearson’s product moment correlation.

### GABA_A_α1 levels are regulated through ubiquitin-proteasomal degradation

**Figure 2:**
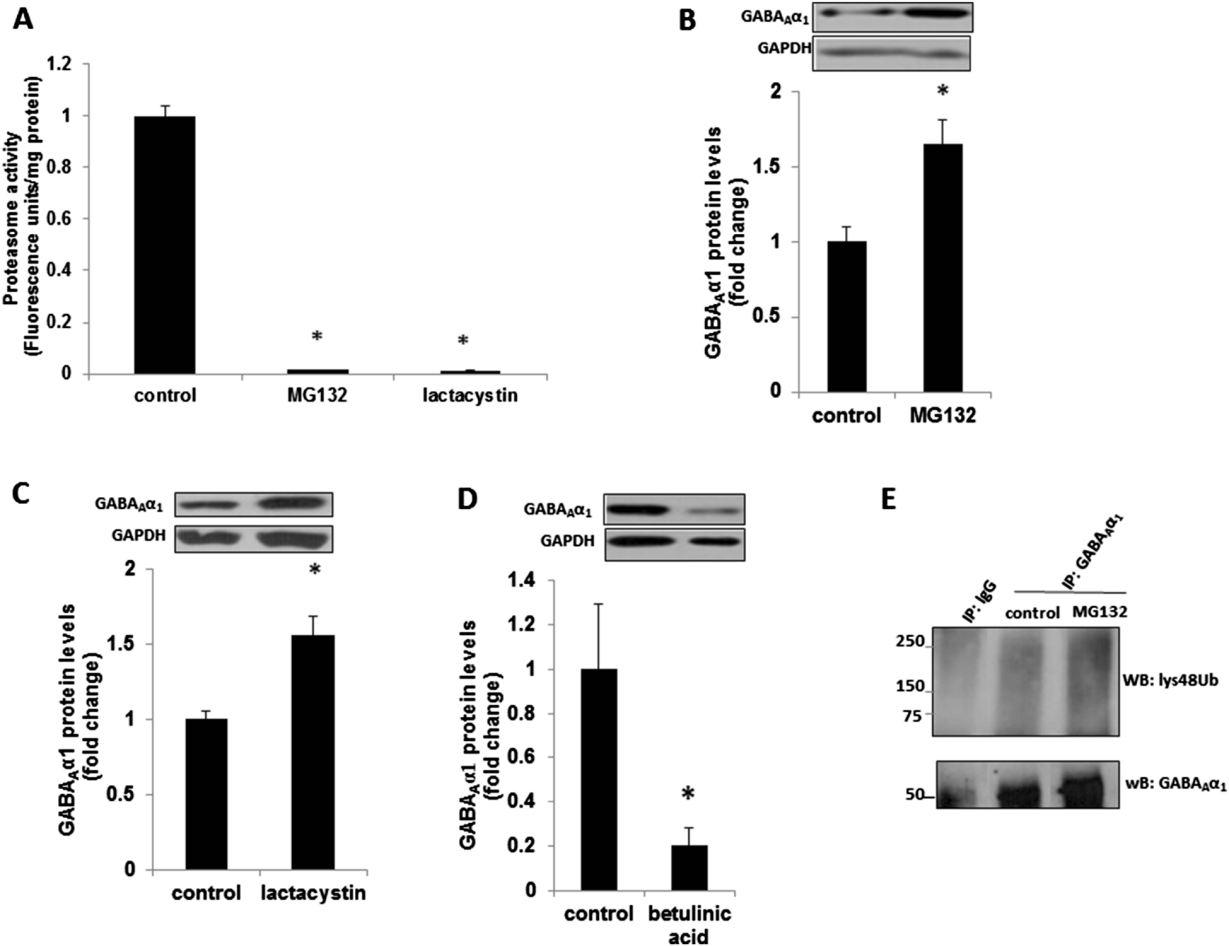
**GABA**_**A**_**α1 levels are regulated through ubiquitin-proteasomal Degradation. (A)** Primary cortical neurons (DIV 14) were treated with vehicle (control; DMSO), MG132 (20 μM), or lactacystin (50 μM) for 9 h, and lysates were subjected to proteasome activity assay. Results are mean ± SEM. *P <0.05 *vs.* control. **(B-D)** Primary cortical neurons (DIV 14) were treated with vehicle (control) or MG132 (20 μM), lactacystin (50 μM), or betulinic acid (20 μg/mL) for 9 h, and lysates were subjected to western blot analysis. The upper panel shows representative autoradiogram of GABA_A_α1 or GAPDH, and the lower panels represent fold change in normalized GABA_A_α1 protein levels in **(B)** MG132, **(C)** lactacystin, or **(C)** butelinic acid-treated neurons. Results are mean ± SEM. *P <0.05 *vs.* control. **(E)** Primary cortical neurons (DIV 14) were treated with vehicle (control) or MG132 (20 μM) for 9 h, and lysates were immunoprecipitated (IP) with an anti-GABA_A_α1 antibody, followed by western blotting (WB) with an anti- ubiquitin Lys48-specific antibody (lys48Ub) or anti-GABA_A_α1 antibody. IgG, IgG control. Results are representative of three independent experiments.

It is known that proteasome-mediated degradation of cellular proteins including molecules involved in neuroplasticity is a critical mechanism for the post-translational regulation of protein turnover [12]. Given that the GABA_A_α1 downregulation in the ASD subjects occurred at the post-translational level, we examined whether proteasomal degradation would play a major role in this process. To examine whether GABA_A_α1s are degraded by proteasomes, we treated cultured primary cortical neurons for 9 h with the proteasomal inhibitors, lactacystin or MG132, and determined proteasome activity and GABA_A_α1 protein levels. We found significant reductions in proteasome activity following treatment with MG132 and lactacystin in neurons (Figure 2A). In addition, both MG132 (Figure 2B) and lactacystin (Figure 2C) significantly increased GABA_A_α1 protein levels as compared to vehicle-treated neurons. Next, we examined whether increasing proteasome activity could decrease GABA_A_α1 protein levels in neurons. We treated cultured primary cortical neurons for 9 h with a proteasome activator, betulinic acid, and determined the GABA_A_α1 protein levels. The treatment with betulinic acid significantly decreased GABA_A_α1 protein levels in cortical neurons (Figure 2D). These findings suggest that proteasomal degradation plays an important role in the regulation of GABA_A_α1 receptors in neurons.

An important step in the proteasomal degradation pathway is the formation of an ubiquitin-protein conjugate [22]. The ubiquitination leads to the covalent binding of ubiquitin ligase to the target molecule and subsequent degradation of the molecule by the 26 S proteasome [23]. Specifically, Lys-48 linked poly-ubiquitination is most commonly associated with proteins targeted for proteasomal degradation [16]. We found an interaction between GABA_A_α1 and Lys48-ubiquitin in cortical neurons suggesting a possible Lys48-linked polyubiquitination of GABA_A_α1 in neurons (Figure 2E). Moreover, treatment with MG132 significantly increased Lys48-linked polyubiquitination of GABA_A_α1 in neurons as determined by immunoprecipitation. Collectively, the preventive effect of proteasome inhibitors on GABA_A_α1 degradation, increased proteasome activity, and Lys48-linked polyubiquitination of GABA_A_α1 indicate that polyubiquitination of GABA_A_α1 proteins with their subsequent proteasomal degradation occurs in cortical neurons.

### Ubiquitination pathway is altered in the middle frontal gyrus of ASD subjects

**Figure 3:**
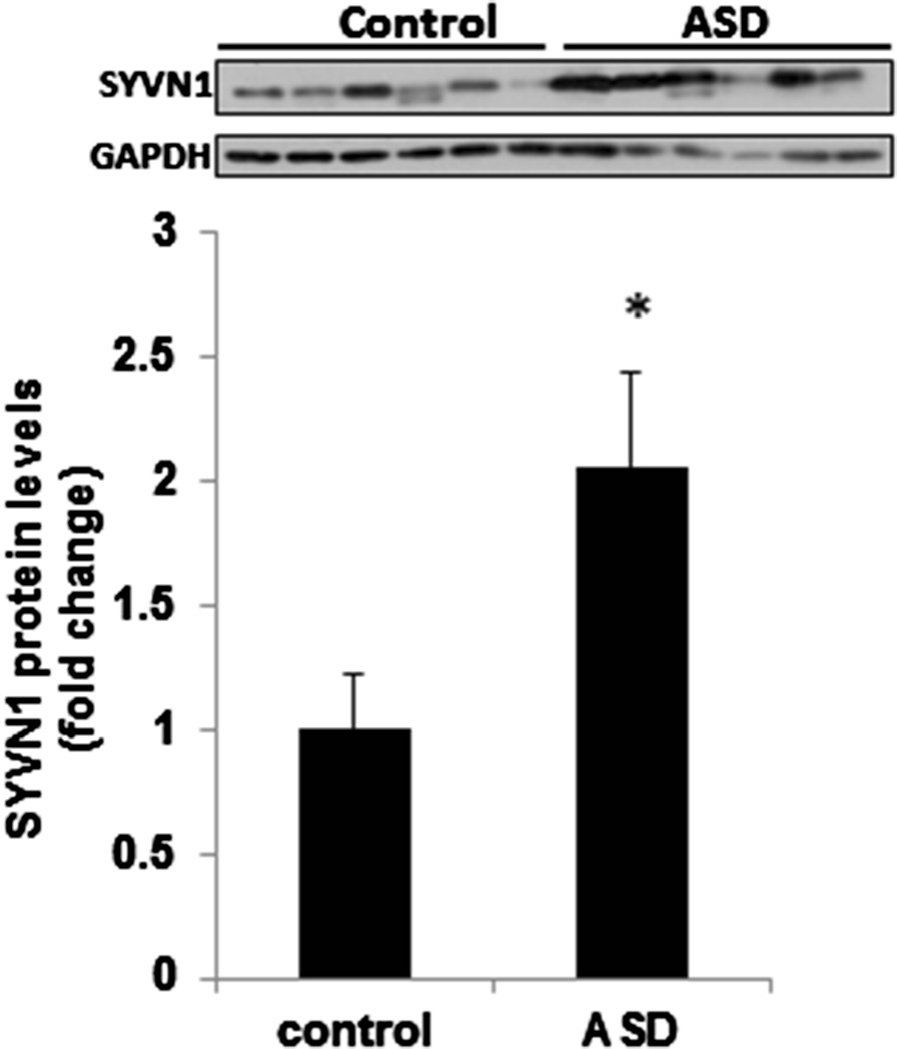
**Increased SYVN1 protein expression in the middle frontal cortex of ASD subjects.** SYVN1 protein levels were determined by western blot analysis. The upper panel shows representative autoradiogram of SYVN1 or GAPDH, and the lower panels represent fold change in normalized SYVN1 protein levels. Results are mean ± SEM. *P <0.05 *vs.* controls.

Since we found a potential role of ubiquitination-mediated proteasomal pathway in GABA_A_α1 regulation, we examined the ubiquitination profile in the postmortem samples from ASD and control subjects using ubiquitination-specific RT-PCR array. The array consisted of 84 genes belonging to E1 (ubiquitin-activating enzyme), E2 (ubiquitin-conjugating enzyme), and E3 (ubiquitin ligase) family. The data were analyzed to the arithmetic mean of three housekeeping genes (HPRT1, RPL13A, and GAPDH). Additional file 3: Table S3 summarizes the fold change and *P* values for the ubiquitin ligases examined in ASD and control subjects. The cutoff fold change for significance in the array was set to ≥2 as per the assay instructions. Accordingly, SYVN1 was the only E3 ligase which showed statistically significant fold difference between ASD and control subjects in the array. The data on SYVN1 from the array were further confirmed using western blot analysis (Figure 3). There was a statistically significant difference between people with ASD (EMM = 1.30, SE = 0.17) and controls (EMM = 0.766, SE = 0.17) in their SYVN1 protein expression with greater increase in SYVN1 in the ASD group (*F*(1, 21) = 4.561, *P* = 0.044, η^2^_p_ = 0.172). Age, postmortem interval, and RNA integrity number were all included in the model as covariates. Age (*F*(1, 21) = 10.73, *P* = 0.003, η^2^_p_ = 0.328) and RNA integrity number (*F*(1, 21) = 6.45, *P* = 0.019, η^2^_p_ = 0.23) were the only significant covariates in the model. The above data indicate that the alterations in the expression of SYVN1 occur at both mRNA and protein levels. We did not find any significant correlation between the protein expression of SYVN1 and the confounding variables, such as age at death, PMI, refrigeration interval, brain pH, or RNA integrity in control or ASD subjects (Table 3). However, a large and statistically significant inverse correlation (r = -0.712; *P* = 0.047) was observed between SYVN1 levels and non-verbal communication.

**Table 3:** Correlations of SYVN1 protein levels with confounding variables

	**CON**		**ASD**	
	**r**	***P***	**r**	***P***
Age	-0.516	0.071	-0.510	0.075
PMI	0.253	0.405	0.271	0.371
Storage	-0.163	0.593	0.227	0.455
RIN	0.240	0.430	0.412	0.173
pH	0.044	0.887	0.437	0.135
Social interaction			0.189	0.626
Verbal communication			-0.580	0.228
Non-verbal communication			-0.712	0.047*
Stereotyped behavior			0.509	0.162
Abnormality of development			-0.319	0.441

Pearson’s product moment correlation.

*P <0.05.

### SYVN1 is the E3 ligase associated with GABA_A_α1

**Figure 4:**
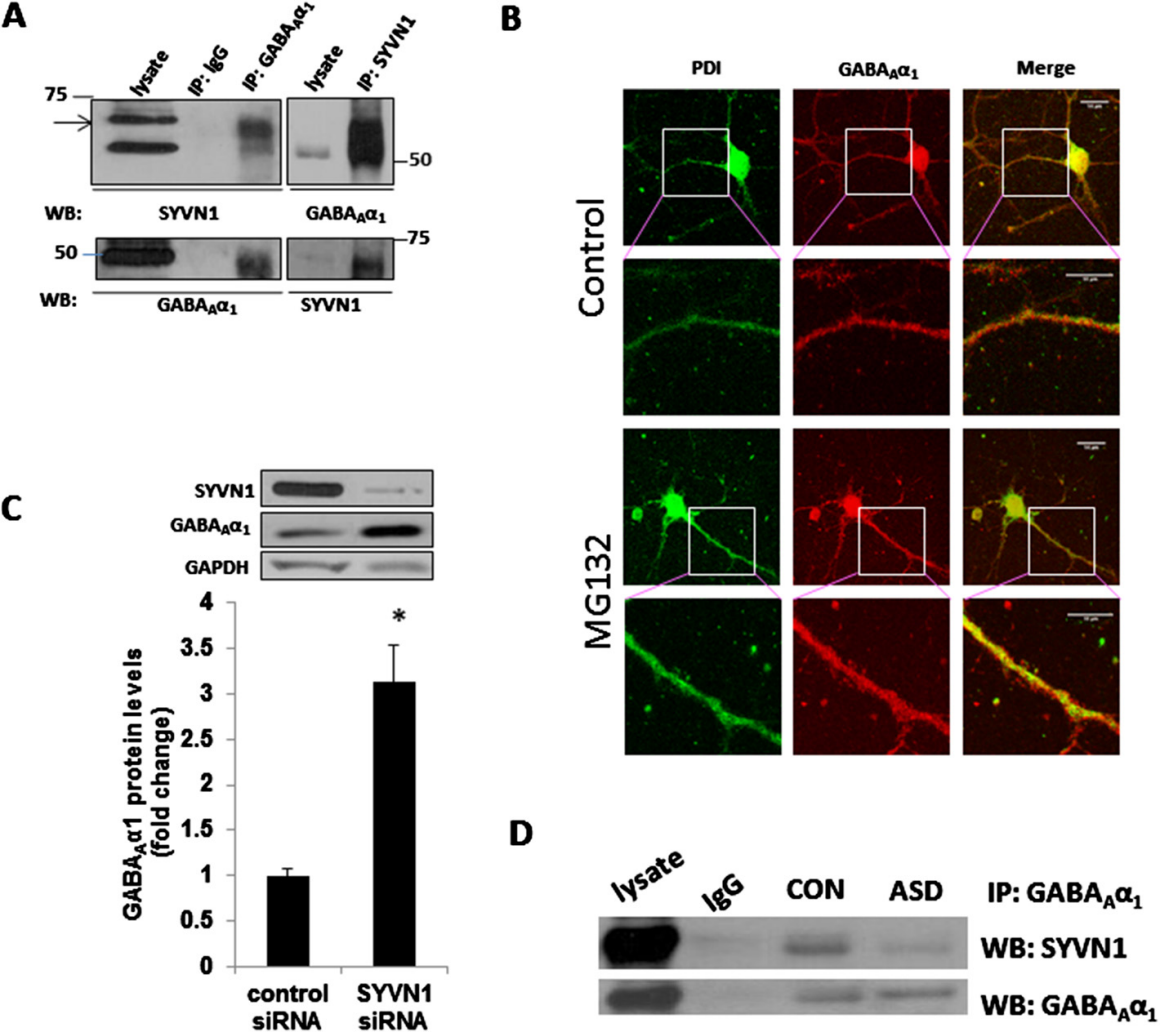
**SYVN1 is the E3 ligase associated with GABA**_**A**_**α1. (A)** GABA_A_α1 co-precipitated with SYVN1. Lysates from primary cortical neurons (DIV 14) were subjected to immunoprecipitation (IP) using an anti-GABA_A_α1 antibody followed by western blotting (WB) with SYVN1 or GABA_A_α1 antibody. In reverse IP, neuronal lysates were immunoprecipiated with an anti-SYVN1 antibody followed by western blotting with GABA_A_α1 or SYVN1 antibody. Lysate represents 10% of the amount used in the IP. IgG, IgG control. Results are representative of three independent experiments. **(B)** Inhibition of proteasomal activity increased the expression of GABA_A_α1 in the ER. Primary cortical neurons (DIV 14) were treated with vehicle (control; DMSO) or MG132 (20 μM) for 9 h, and the co-localization of GABA_A_α1 and ER marker (PDI) was examined by immunofluorescence method. **(C)** SYVN1 knockdown increased GABA_A_α1 protein levels in cortical neurons. Primary cortical neurons (DIV 14) were transfected with control siRNA or SYVN1siRNA, and the protein levels of SYVN1 and GABA_A_α1 were examined at 48 h after transfection. The upper panel shows representative autoradiogram of SYVN1, GABA_A_α1, and GAPDH, and the lower panel represents fold change in normalized GABA_A_α1 protein levels. Results are mean ± SE for at least three independent neuronal preparations. **(D)** Reduced interaction between GABA_A_α1 with SYVN1 in ASD subjects. Lysates from middle frontal gyrus of ASD and control subjects were subjected to IP using an anti-GABA_A_α1 antibody followed by WB with SYVN1 or GABA_A_α1 antibody. Lysate represents 10% of the amount used in the IP. IgG, IgG control.

We sought to examine whether SYVN1 is the E3 ligase partner of GABA_A_α1 responsible for the GABA_A_α1 ubiquitination and its proteasomal degradation. We performed immunoprecipitation assay to determine possible GABA_A_α1 conjugation with SYVN1 in mouse primary cortical neurons, and each of these immunoprecipitates was examined for co-purification of SYVN1 by western blot. SYVN1 was detected in GABA_A_α1 immunoprecipitates, but not in the control IgG (Figure 4A). The above interaction was further confirmed using a reciprocal approach, where GABA_A_α1 co-purified with SYVN1, but not with a control IgG. Since SYVN1 is a known ER-associated degradation (ERAD) E3 ubiquitin ligase, we next examined whether inhibiting the proteasomal activity would lead to the accumulation of GABA_A_α1 in ER. MG132 significantly increased the expression of GABA_A_α1 in the ER as evidenced by the increase in the complex between GABA_A_α1 and ER marker, PDI (Figure 4B).

Next, we examined the causal relationship between SYVN1 and GABA_A_α1 degradation by using SYVN1 siRNA in neurons. Primary cortical neurons were treated with siCONTROL or the SYVN1 siRNA, and the expression of SYVN1 and GABA_A_α1 protein levels were determined. The SYVN1 expression level was significantly reduced in those cells transfected with the SYVN1 siRNA when examined 48 h after the transfection. The siRNA knockdown of SYVN1 significantly increased GABA_A_α1 protein levels, as compared with the siCONTROL transfectants (*P* <0.05; Figure 4C). We performed immunoprecipitation assay to determine whether the association between GABA_A_α1 and SYVN1 is altered in ASD. We found a reduced interaction between GABA_A_α1 and SYVN1 in the middle frontal gyrus of ASD subjects as compared to controls (Figure 4D). Taken together, these results indicate that SYVN1 plays a critical role as an E3 ligase in the UPS-mediated GABA_A_α1 degradation.

## Discussion

The findings from the present study demonstrate a UPS-mediated mechanism critical for the post-translational regulation of the GABA_A_α1. In primary cortical neurons, the UPS-mediated GABA_A_α1 degradation contributed to the physiological GABA_A_α1 turnover because inhibition of the proteasome activity improved the basal level of endogenous GABA_A_α1 levels. The UPS-mediated GABA_A_α1 turnover was also substantially enhanced in the cortical samples from ASD subjects resulting in altered GABA_A_α1 levels. Because the GABAergic system plays an important role in a variety of cellular functions including neuroplasticity, preventing an excessive GABA_A_α1 turnover may be an important mechanism in maintaining GABA_A_α1 levels and GABA signaling.

Our data show that the change in GABA_A_α1 expression in ASD occurs at the post-translational level. Although an earlier study has reported decrease in GABA_A_α1 protein levels in the frontal cortex of autism [11], the receptor status at the mRNA level has never been examined before. Given that UPS plays an important role in the regulation of receptors, we examined the role of UPS activity in the downregulation of GABA_A_α1 using postmortem brain samples as well as mouse primary cortical neurons. Our data demonstrate that the expression of SYVN1 was higher in the tissue samples from ASD as compared to controls. Moreover, treatment with proteasomal inhibitors as well as inhibition of E3 ubiquitin ligase (SYVN1) significantly increased GABA_A_α1 protein levels in cortical neurons. These results indicate that the degradation of GABA_A_α1 may be subject to proteasomal regulation through a SYVN1-mediated cellular pathway.

GABA_A_ receptors play important roles in various neurodevelopmental processes including proliferation, migration, and differentiation of precursor cells [7-10]. The α1 subunit receptors appear to be responsible for sedative effects of positive allosteric modulators of the GABA_A_ system, such as diazepam [6]. Moreover, reductions in GABA_A_ receptor binding have been found in the hippocampus [24] and anterior and posterior cingulate cortex [25,26] of ASD subjects. The above changes in receptor densities/affinities have also been reflected in receptor expression levels. GABA_A_α1 protein levels were significantly lower in the superior frontal cortex and parietal cortex of subjects with autism [11]. However, the mechanisms of regulation of GABA_A_α1 in ASD were not clearly understood. GABA_A_ receptor subunits are inserted co-translationally into the membrane of the ER and oligomerized in the presence of ER-resident chaperones. The ERAD plays an important role in the degradation of un- or misfolded GABA receptors [27,28]. Accordingly, a recent study has indicated that even relatively low levels of ER stress may alter the membrane trafficking of the synaptic functional molecules such as GABA receptors leading to ASD pathophysiology [29]. It has been shown that the ubiquitination of GABA_A_α1 targets the unassembled subunits within the ER for proteasome-dependent degradation [28]. The increase in GABA_A_α1 protein levels following proteasomal inhibition in the present study supports the above findings. Moreover, we identified the SYVN1 as the ERAD E3 ubiquitin ligase involved in GABA_A_α1 regulation using *in vitro* neurons. Interestingly, SYVN1 has recently been reported in the regulation of GABA_B_ receptors [14] suggesting that ERAD plays an important role in the control of functional GABA receptors and their trafficking.

## Conclusions

The findings from the present study may have functional implications in the cellular mechanisms of ASD pathophysiology. Recent studies have shed light on the neurobiology of ASD including those related to the GABAergic system [2] and ER stress [29]. The present data suggest a possible link between regulation of GABA_A_α1 turnover and the ERAD-mediated proteasomal degradation pathway. The above relationship should be further investigated *in vivo* using an appropriate animal model for autism such as fragile-X knockout or BTBR mouse. In addition, the current findings represent only one brain region whereas abnormalities in GABAergic function have been reported in many other brain regions including hippocampus in ASD. Therefore, additional studies should investigate the role of proteasomal degradation pathway in GABA_A_α1 regulation in other brain regions implicated in ASD. The present data may have potential clinical implications. For example, using proteasome inhibitor and/or targeting key elements of the UPS-mediated GABA_A_α1 turnover (for example, SYVN1) could offer a new strategy for treating GABAergic deficits often seen in ASD and related CNS disorders.

## Availability of supporting data

The datasets supporting the results of this article are included within the article and its additional files.

## Abbreviations

ADI-R: Autism diagnostic interview revised; ASD: Autism spectrum disorders; ERAD: Endoplasmic-reticulum-associated protein degradation GABA, γ-amino butyric acid; SYVN1: Synovial apoptosis inhibitor 1; UPS: Ubiquitin-proteasome system.

## Competing interests

The authors declare that they have no competing interests.

## Authors’ contributions

AC carried out the gene as well as protein expression studies, and drafted the manuscript. CDP and DP performed the immunoprecipitation and immunofluorescence studies, and helped to draft the manuscript. AOA carried out the statistical analysis and helped to revise the manuscript. AP conceived of the study, and participated in its design and coordination and wrote the manuscript. All authors read and approved the final manuscript.

## Supplementary Material

Additional file 1: Table S1 Postmortem brain tissue information. Click here for file

Additional file 2: Table S2 Descriptive statistics of study variables. Click here for file

Additional file 3: Table S3 Ubiquitination array data. Click here for file
