# Supplementary material for: Ubiquitin-proteasome dependent degradation of GABAAα1 in autism spectrum disorder
Source: Mol Autism. 2014 Sep 1;5:45. doi: 10.1186/2040-2392-5-45 (PMC4228821; doi:10.1186/2040-2392-5-45)
Supplement: Additional file 1: Table S1 — Postmortem brain tissue information. [file 2040-2392-5-45-S1.docx]

| **UMB #** | **Group** | **PMI**  **(h)** | **Storage (days)** | **Ethnicity** | **Medication history** | **Cause of death** |
| --- | --- | --- | --- | --- | --- | --- |
| 3 | Control | 17 | 7,764 | Caucasian |  | Accident, drowning |
| 142 | Control | 7 | 5,777 | Caucasian |  | Accident, head injuries |
| 229 | Control | 19 | 7,042 | Caucasian |  | Accident, hanging |
| 356 | Control | 14 | 7,158 | African American |  | MV accident, multiple injuries |
| 1158 | Control | 15 | 4,792 | Caucasian |  | Cardiomegaly |
| 1475 | Control | 3 | 4,598 | Caucasian |  | Multiple injuries (car accident) |
| 1674 | Control | 36 | 3,830 | Caucasian |  | Hypothermia and drowning |
| 4337 | Control | 16 | 1,569 | African American |  | Blunt force neck injury, MVA |
| 4672 | Control | 14 | 2,924 | African American |  | Anaphalactoid reaction |
| 4898 | Control | 12 | 2,906 | Caucasian | Concerta, clonidone | Drowning |
| 5309 | Control | 8 | 1,770 | Caucasian |  | TSS |
| 5334 | Control | 15 | 452 | Hispanic |  | Hanging/Suicide |
| M3415M | Control | 12 | 5,147 | African American |  | Multisystem failure |
|  |  |  |  |  |  |  |
| 797 | ASD/Autism | 13 | 6,182 | Caucasian |  | Drowning |
| 1349 | ASD/Autism | 39 | 4,465 | Caucasian |  | Drowning |
| 4231 | ASD/Autism | 12 | 3,272 | African American | Zyprexa, Reminyl | Drowning |
| 4334 | ASD/Autism | 27 | 1,626 | Hispanic |  | Acute hemorrhagic tracheobronchitis |
| 4721 | ASD/Autism | 16 | 2,931 | African American |  | Drowning |
| 4849 | ASD/Autism | 20 | 2,615 | African American |  | Drowning |
| 4899 | ASD/Autism | 9 | 2,555 | Caucasian |  | Drowning |
| 4999 | ASD/Autism | 14 | 2,029 | Caucasian | Naltrexone | Cardiac arrhythmia |
| 5144 | ASD/Autism | 3 | 1,940 | Caucasian |  | Rhabdomyosarcoma |
| 5176 | ASD/Autism | 18 | 1,848 | African American | Risperdal | Subdural hemorrhage |
| 5302 | ASD/Autism | 20 | 4,426 | Caucasian | Risperdal, Luvox, Clonidine, Insulin | Diabetic ketoacidosis |
| 5308 | ASD/Autism | 21 | 1,756 | Caucasian |  | Skull fractures, struck by car |
| 5403 | ASD/Autism | 35 | 1,129 | Caucasian |  | Cardiac arrhythmia |

**Table S1. Postmortem brain tissue information.**
