# Supplementary material for: Ubiquitin-proteasome dependent degradation of GABAAα1 in autism spectrum disorder
Source: Mol Autism. 2014 Sep 1;5:45. doi: 10.1186/2040-2392-5-45 (PMC4228821; doi:10.1186/2040-2392-5-45)
Supplement: Additional file 2: Table S2 — Descriptive statistics of study variables. [file 2040-2392-5-45-S2.docx]

**Table S2.** **Descriptive statistics of study variables.**

| **Variable** | **Mean** | | **SD** | **Skew** | **Kurtosis** |
| --- | --- | --- | --- | --- | --- |
| Age (years) | | 11.75 | 5.64 | 0.57 | -0.90 |
| Postmortem interval (hours) | | 16.73 | 9.10 | 1.03 | 1.04 |
| Storage time (days) | | 3,557.81 | 2,021.23 | 0.60 | -0.62 |
| Sample pH | | 6.03 | 0.25 | -0.43 | 0.79 |
| RNA integrity number | | 6.19 | 2.31 | -0.45 | -1.29 |
| GABA_A_α1 mRNA | | 8.38 | 2.62 | 0.51 | 0.63 |
| GABA_A_α1 protein | | 0.82 | 0.41 | 1.00 | 0.43 |
| SYVN1 protein | | 1.04 | 0.84 | 0.99 | -0.09 |
| Social interaction | | 25.33 | 5.45 | 0.67 | 1.04 |
| Verbal communication | | 15.67 | 4.41 | -0.51 | -1.14 |
| Non-verbal communication | | 12.38 | 1.92 | -1.03 | -0.31 |
| Stereotyped behavior | | 6.33 | 2.18 | 0.10 | -0.39 |
| Abnormality of development | | 3.75 | 0.89 | 0.62 | -1.48 |
