# Supplementary material for: Ubiquitin-proteasome dependent degradation of GABAAα1 in autism spectrum disorder
Source: Mol Autism. 2014 Sep 1;5:45. doi: 10.1186/2040-2392-5-45 (PMC4228821; doi:10.1186/2040-2392-5-45)
Supplement: Additional file 3: Table S3 — Ubiquitination array data. [file 2040-2392-5-45-S3.docx]

**Table S3. Ubiquitination array data.**

| **Accession #** | **Symbol** | **Description** | ***P* value** | **Fold regulation (ASD/Controls)** |
| --- | --- | --- | --- | --- |
| [NM_001002244](http://www.ncbi.nlm.nih.gov/sites/entrez?db=gene&cmd=Retrieve&dopt=Graphics&list_uids=51529) | ANAPC11 | Anaphase promoting complex subunit 11 | 0.284393 | -1.08 |
| [NM_013366](http://www.ncbi.nlm.nih.gov/sites/entrez?db=gene&cmd=Retrieve&dopt=Graphics&list_uids=29882) | ANAPC2 | Anaphase promoting complex subunit 2 | 0.111110 | 1.44 |
| [NM_005744](http://www.ncbi.nlm.nih.gov/sites/entrez?db=gene&cmd=Retrieve&dopt=Graphics&list_uids=25820) | ARIH1 | Ariadne homolog, ubiquitin-conjugating enzyme E2 binding protein, 1 (Drosophila) | 0.651089 | 1.01 |
| [NM_006395](http://www.ncbi.nlm.nih.gov/sites/entrez?db=gene&cmd=Retrieve&dopt=Graphics&list_uids=10533) | ATG7 | ATG7 autophagy related 7 homolog (S. cerevisiae) | 0.977005 | 1.01 |
| [NM_000465](http://www.ncbi.nlm.nih.gov/sites/entrez?db=gene&cmd=Retrieve&dopt=Graphics&list_uids=580) | BARD1 | BRCA1 associated RING domain 1 | 0.628144 | 1.05 |
| [NM_007294](http://www.ncbi.nlm.nih.gov/sites/entrez?db=gene&cmd=Retrieve&dopt=Graphics&list_uids=672) | BRCA1 | Breast cancer 1, early onset | 0.715447 | 1.11 |
| [NM_024332](http://www.ncbi.nlm.nih.gov/sites/entrez?db=gene&cmd=Retrieve&dopt=Graphics&list_uids=79184) | BRCC3 | BRCA1/BRCA2-containing complex, subunit 3 | 0.397974 | -1.11 |
| [NM_033637](http://www.ncbi.nlm.nih.gov/sites/entrez?db=gene&cmd=Retrieve&dopt=Graphics&list_uids=8945) | BTRC | Beta-transducin repeat containing | 0.485166 | -1.06 |
| [NM_005188](http://www.ncbi.nlm.nih.gov/sites/entrez?db=gene&cmd=Retrieve&dopt=Graphics&list_uids=867) | CBL | Cas-Br-M (murine) ecotropic retroviral transforming sequence | 0.078257 | 1.52 |
| [NM_004359](http://www.ncbi.nlm.nih.gov/sites/entrez?db=gene&cmd=Retrieve&dopt=Graphics&list_uids=997) | CDC34 | Cell division cycle 34 homolog (S. cerevisiae) | 0.029281 | 1.72 |
| [NM_003592](http://www.ncbi.nlm.nih.gov/sites/entrez?db=gene&cmd=Retrieve&dopt=Graphics&list_uids=8454) | CUL1 | Cullin 1 | 0.081865 | -1.2 |
| [NM_003591](http://www.ncbi.nlm.nih.gov/sites/entrez?db=gene&cmd=Retrieve&dopt=Graphics&list_uids=8453) | CUL2 | Cullin 2 | 0.158222 | -1.22 |
| [NM_003590](http://www.ncbi.nlm.nih.gov/sites/entrez?db=gene&cmd=Retrieve&dopt=Graphics&list_uids=8452) | CUL3 | Cullin 3 | 0.042310 | -1.2 |
| [NM_003589](http://www.ncbi.nlm.nih.gov/sites/entrez?db=gene&cmd=Retrieve&dopt=Graphics&list_uids=8451) | CUL4A | Cullin 4A | 0.389893 | 1.08 |
| [NM_003588](http://www.ncbi.nlm.nih.gov/sites/entrez?db=gene&cmd=Retrieve&dopt=Graphics&list_uids=8450) | CUL4B | Cullin 4B | 0.867060 | -1.04 |
| [NM_003478](http://www.ncbi.nlm.nih.gov/sites/entrez?db=gene&cmd=Retrieve&dopt=Graphics&list_uids=8065) | CUL5 | Cullin 5 | 0.922679 | -1.03 |
| [NM_014780](http://www.ncbi.nlm.nih.gov/sites/entrez?db=gene&cmd=Retrieve&dopt=Graphics&list_uids=9820) | CUL7 | Cullin 7 | 0.841168 | -1.04 |
| [NM_015089](http://www.ncbi.nlm.nih.gov/sites/entrez?db=gene&cmd=Retrieve&dopt=Graphics&list_uids=23113) | CUL9 | Cullin 9 | 0.024904 | 1.75 |
| [NM_001923](http://www.ncbi.nlm.nih.gov/sites/entrez?db=gene&cmd=Retrieve&dopt=Graphics&list_uids=1642) | DDB1 | Damage-specific DNA binding protein 1, 127kDa | 0.027974 | 1.17 |
| [NM_014648](http://www.ncbi.nlm.nih.gov/sites/entrez?db=gene&cmd=Retrieve&dopt=Graphics&list_uids=9666) | DZIP3 | DAZ interacting protein 3, zinc finger | 0.552761 | -1.05 |
| [NM_012175](http://www.ncbi.nlm.nih.gov/sites/entrez?db=gene&cmd=Retrieve&dopt=Graphics&list_uids=26273) | FBXO3 | F-box protein 3 | 0.708055 | -1.17 |
| [NM_024735](http://www.ncbi.nlm.nih.gov/sites/entrez?db=gene&cmd=Retrieve&dopt=Graphics&list_uids=79791) | FBXO31 | F-box protein 31 | 0.370229 | 1.14 |
| [NM_012176](http://www.ncbi.nlm.nih.gov/sites/entrez?db=gene&cmd=Retrieve&dopt=Graphics&list_uids=26272) | FBXO4 | F-box protein 4 | 0.863153 | -1.05 |
| [NM_031456](http://www.ncbi.nlm.nih.gov/sites/entrez?db=gene&cmd=Retrieve&dopt=Graphics&list_uids=10517) | FBXW10 | F-box and WD repeat domain containing 10 | 0.892869 | -1.26 |
| [NM_032301](http://www.ncbi.nlm.nih.gov/sites/entrez?db=gene&cmd=Retrieve&dopt=Graphics&list_uids=84261) | FBXW9 | F-box and WD repeat domain containing 9 | 0.347865 | -1.1 |
| [NM_015052](http://www.ncbi.nlm.nih.gov/sites/entrez?db=gene&cmd=Retrieve&dopt=Graphics&list_uids=23072) | HECW1 | HECT, C2 and WW domain containing E3 ubiquitin protein ligase 1 | 0.292170 | -1.14 |
| [NM_020760](http://www.ncbi.nlm.nih.gov/sites/entrez?db=gene&cmd=Retrieve&dopt=Graphics&list_uids=57520) | HECW2 | HECT, C2 and WW domain containing E3 ubiquitin protein ligase 2 | 0.605351 | 1.17 |
| [NM_016323](http://www.ncbi.nlm.nih.gov/sites/entrez?db=gene&cmd=Retrieve&dopt=Graphics&list_uids=51191) | HERC5 | Hect domain and RLD 5 | 0.196287 | 1.43 |
| [NM_031407](http://www.ncbi.nlm.nih.gov/sites/entrez?db=gene&cmd=Retrieve&dopt=Graphics&list_uids=10075) | HUWE1 | HECT, UBA and WWE domain containing 1 | 0.082444 | 1.15 |
| [NM_017824](http://www.ncbi.nlm.nih.gov/sites/entrez?db=gene&cmd=Retrieve&dopt=Graphics&list_uids=54708) | MARCH5 | Membrane-associated ring finger (C3HC4) 5 | 0.570201 | -1.09 |
| [NM_002392](http://www.ncbi.nlm.nih.gov/sites/entrez?db=gene&cmd=Retrieve&dopt=Graphics&list_uids=4193) | MDM2 | Mdm2 p53 binding protein homolog (mouse) | 0.885450 | -1.01 |
| [NM_020774](http://www.ncbi.nlm.nih.gov/sites/entrez?db=gene&cmd=Retrieve&dopt=Graphics&list_uids=57534) | MIB1 | Mindbomb homolog 1 (Drosophila) | 0.763448 | 1.02 |
| [NM_014484](http://www.ncbi.nlm.nih.gov/sites/entrez?db=gene&cmd=Retrieve&dopt=Graphics&list_uids=27304) | MOCS3 | Molybdenum cofactor synthesis 3 | 0.912374 | 1.01 |
| [NM_024544](http://www.ncbi.nlm.nih.gov/sites/entrez?db=gene&cmd=Retrieve&dopt=Graphics&list_uids=79594) | MUL1 | Mitochondrial E3 ubiquitin protein ligase 1 | 0.363669 | 2.03 |
| [NM_003905](http://www.ncbi.nlm.nih.gov/sites/entrez?db=gene&cmd=Retrieve&dopt=Graphics&list_uids=8883) | NAE1 | NEDD8 activating enzyme E1 subunit 1 | 0.149022 | -1.34 |
| [NM_006156](http://www.ncbi.nlm.nih.gov/sites/entrez?db=gene&cmd=Retrieve&dopt=Graphics&list_uids=4738) | NEDD8 | Neural precursor cell expressed, developmentally down-regulated 8 | 0.632159 | -1.02 |
| [NM_004562](http://www.ncbi.nlm.nih.gov/sites/entrez?db=gene&cmd=Retrieve&dopt=Graphics&list_uids=5071) | PARK2 | Parkinson protein 2, E3 ubiquitin protein ligase (parkin) | 0.419020 | -1.06 |
| [NM_022457](http://www.ncbi.nlm.nih.gov/sites/entrez?db=gene&cmd=Retrieve&dopt=Graphics&list_uids=64326) | RFWD2 | Ring finger and WD repeat domain 2 | 0.617572 | 1.36 |
| [NM_022064](http://www.ncbi.nlm.nih.gov/sites/entrez?db=gene&cmd=Retrieve&dopt=Graphics&list_uids=63891) | RNF123 | Ring finger protein 123 | 0.046439 | 1.85 |
| [NM_198085](http://www.ncbi.nlm.nih.gov/sites/entrez?db=gene&cmd=Retrieve&dopt=Graphics&list_uids=378925) | RNF148 | Ring finger protein 148 | 0.287921 | -1.46 |
| [NM_005500](http://www.ncbi.nlm.nih.gov/sites/entrez?db=gene&cmd=Retrieve&dopt=Graphics&list_uids=10055) | SAE1 | SUMO1 activating enzyme subunit 1 | 0.850785 | -1.07 |
| [NM_006930](http://www.ncbi.nlm.nih.gov/sites/entrez?db=gene&cmd=Retrieve&dopt=Graphics&list_uids=6500) | SKP1 | S-phase kinase-associated protein 1 | 0.059159 | -1.28 |
| [NM_005983](http://www.ncbi.nlm.nih.gov/sites/entrez?db=gene&cmd=Retrieve&dopt=Graphics&list_uids=6502) | SKP2 | S-phase kinase-associated protein 2 (p45) | 0.872348 | -1.02 |
| [NM_020429](http://www.ncbi.nlm.nih.gov/sites/entrez?db=gene&cmd=Retrieve&dopt=Graphics&list_uids=57154) | SMURF1 | SMAD specific E3 ubiquitin protein ligase 1 | 0.842290 | 1.08 |
| [NM_022739](http://www.ncbi.nlm.nih.gov/sites/entrez?db=gene&cmd=Retrieve&dopt=Graphics&list_uids=64750) | SMURF2 | SMAD specific E3 ubiquitin protein ligase 2 | 0.071562 | -1.4 |
| [NM_005861](http://www.ncbi.nlm.nih.gov/sites/entrez?db=gene&cmd=Retrieve&dopt=Graphics&list_uids=10273) | STUB1 | STIP1 homology and U-box containing protein 1, E3 ubiquitin protein ligase | 0.115082 | 1.25 |
| [NM_172230](http://www.ncbi.nlm.nih.gov/sites/entrez?db=gene&cmd=Retrieve&dopt=Graphics&list_uids=84447) | SYVN1 | Synovial apoptosis inhibitor 1, synoviolin | 0.007401 | 2 |
| [NM_199129](http://www.ncbi.nlm.nih.gov/sites/entrez?db=gene&cmd=Retrieve&dopt=Graphics&list_uids=387521) | TMEM189 | Transmembrane protein 189 | 0.119141 | 1.27 |
| [NM_000546](http://www.ncbi.nlm.nih.gov/sites/entrez?db=gene&cmd=Retrieve&dopt=Graphics&list_uids=7157) | TP53 | Tumor protein p53 | 0.307222 | 1.31 |
| [NM_003334](http://www.ncbi.nlm.nih.gov/sites/entrez?db=gene&cmd=Retrieve&dopt=Graphics&list_uids=7317) | UBA1 | Ubiquitin-like modifier activating enzyme 1 | 0.007952 | 1.29 |
| [NM_005499](http://www.ncbi.nlm.nih.gov/sites/entrez?db=gene&cmd=Retrieve&dopt=Graphics&list_uids=10054) | UBA2 | Ubiquitin-like modifier activating enzyme 2 | 0.242749 | -1.14 |
| [NM_003968](http://www.ncbi.nlm.nih.gov/sites/entrez?db=gene&cmd=Retrieve&dopt=Graphics&list_uids=9039) | UBA3 | Ubiquitin-like modifier activating enzyme 3 | 0.887893 | -1.02 |
| [NM_198329](http://www.ncbi.nlm.nih.gov/sites/entrez?db=gene&cmd=Retrieve&dopt=Graphics&list_uids=79876) | UBA5 | Ubiquitin-like modifier activating enzyme 5 | 0.040568 | -1.27 |
| [NM_018227](http://www.ncbi.nlm.nih.gov/sites/entrez?db=gene&cmd=Retrieve&dopt=Graphics&list_uids=55236) | UBA6 | Ubiquitin-like modifier activating enzyme 6 | 0.970984 | 1.03 |
| [NM_003336](http://www.ncbi.nlm.nih.gov/sites/entrez?db=gene&cmd=Retrieve&dopt=Graphics&list_uids=7319) | UBE2A | Ubiquitin-conjugating enzyme E2A | 0.315559 | -1.12 |
| [NM_003337](http://www.ncbi.nlm.nih.gov/sites/entrez?db=gene&cmd=Retrieve&dopt=Graphics&list_uids=7320) | UBE2B | Ubiquitin-conjugating enzyme E2B | 0.031691 | -1.25 |
| [NM_181803](http://www.ncbi.nlm.nih.gov/sites/entrez?db=gene&cmd=Retrieve&dopt=Graphics&list_uids=11065) | UBE2C | Ubiquitin-conjugating enzyme E2C | 0.382417 | -1.21 |
| [NM_003338](http://www.ncbi.nlm.nih.gov/sites/entrez?db=gene&cmd=Retrieve&dopt=Graphics&list_uids=7321) | UBE2D1 | Ubiquitin-conjugating enzyme E2D 1 | 0.083562 | -1.21 |
| [NM_181838](http://www.ncbi.nlm.nih.gov/sites/entrez?db=gene&cmd=Retrieve&dopt=Graphics&list_uids=7322) | UBE2D2 | Ubiquitin-conjugating enzyme E2D 2 | 0.407902 | -1.29 |
| [NM_181893](http://www.ncbi.nlm.nih.gov/sites/entrez?db=gene&cmd=Retrieve&dopt=Graphics&list_uids=7323) | UBE2D3 | Ubiquitin-conjugating enzyme E2D 3 | 0.209208 | -1.14 |
| [NM_182666](http://www.ncbi.nlm.nih.gov/sites/entrez?db=gene&cmd=Retrieve&dopt=Graphics&list_uids=7324) | UBE2E1 | Ubiquitin-conjugating enzyme E2E 1 | 0.877852 | -1.01 |
| [NM_152653](http://www.ncbi.nlm.nih.gov/sites/entrez?db=gene&cmd=Retrieve&dopt=Graphics&list_uids=7325) | UBE2E2 | Ubiquitin-conjugating enzyme E2E 2 | 0.278533 | 1.14 |
| [NM_006357](http://www.ncbi.nlm.nih.gov/sites/entrez?db=gene&cmd=Retrieve&dopt=Graphics&list_uids=10477) | UBE2E3 | Ubiquitin-conjugating enzyme E2E 3 | 0.327820 | -1.07 |
| [NM_003342](http://www.ncbi.nlm.nih.gov/sites/entrez?db=gene&cmd=Retrieve&dopt=Graphics&list_uids=7326) | UBE2G1 | Ubiquitin-conjugating enzyme E2G 1 | 0.328525 | 1.15 |
| [NM_182688](http://www.ncbi.nlm.nih.gov/sites/entrez?db=gene&cmd=Retrieve&dopt=Graphics&list_uids=7327) | UBE2G2 | Ubiquitin-conjugating enzyme E2G 2 | 0.827656 | -1.22 |
| [NM_182697](http://www.ncbi.nlm.nih.gov/sites/entrez?db=gene&cmd=Retrieve&dopt=Graphics&list_uids=7328) | UBE2H | Ubiquitin-conjugating enzyme E2H | 0.805120 | -1.01 |
| [NM_003345](http://www.ncbi.nlm.nih.gov/sites/entrez?db=gene&cmd=Retrieve&dopt=Graphics&list_uids=7329) | UBE2I | Ubiquitin-conjugating enzyme E2I | 0.124922 | 1.17 |
| [NM_016021](http://www.ncbi.nlm.nih.gov/sites/entrez?db=gene&cmd=Retrieve&dopt=Graphics&list_uids=51465) | UBE2J1 | Ubiquitin-conjugating enzyme E2, J1, U | 0.786814 | 1.04 |
| [NM_194458](http://www.ncbi.nlm.nih.gov/sites/entrez?db=gene&cmd=Retrieve&dopt=Graphics&list_uids=118424) | UBE2J2 | Ubiquitin-conjugating enzyme E2, J2 | 0.463420 | 1.07 |
| [NM_005339](http://www.ncbi.nlm.nih.gov/sites/entrez?db=gene&cmd=Retrieve&dopt=Graphics&list_uids=3093) | UBE2K | Ubiquitin-conjugating enzyme E2K | 0.405400 | -1.09 |
| [NM_003347](http://www.ncbi.nlm.nih.gov/sites/entrez?db=gene&cmd=Retrieve&dopt=Graphics&list_uids=7332) | UBE2L3 | Ubiquitin-conjugating enzyme E2L 3 | 0.127723 | -1.15 |
| [NM_003969](http://www.ncbi.nlm.nih.gov/sites/entrez?db=gene&cmd=Retrieve&dopt=Graphics&list_uids=9040) | UBE2M | Ubiquitin-conjugating enzyme E2M | 0.534888 | 1.09 |
| [NM_003348](http://www.ncbi.nlm.nih.gov/sites/entrez?db=gene&cmd=Retrieve&dopt=Graphics&list_uids=7334) | UBE2N | Ubiquitin-conjugating enzyme E2N | 0.580770 | 1.04 |
| [NM_017582](http://www.ncbi.nlm.nih.gov/sites/entrez?db=gene&cmd=Retrieve&dopt=Graphics&list_uids=55585) | UBE2Q1 | Ubiquitin-conjugating enzyme E2Q family member 1 | 0.446662 | 1.06 |
| [NM_017811](http://www.ncbi.nlm.nih.gov/sites/entrez?db=gene&cmd=Retrieve&dopt=Graphics&list_uids=54926) | UBE2R2 | Ubiquitin-conjugating enzyme E2R 2 | 0.495968 | 1.1 |
| [NM_014501](http://www.ncbi.nlm.nih.gov/sites/entrez?db=gene&cmd=Retrieve&dopt=Graphics&list_uids=27338) | UBE2S | Ubiquitin-conjugating enzyme E2S | 0.025902 | 1.81 |
| [NM_014176](http://www.ncbi.nlm.nih.gov/sites/entrez?db=gene&cmd=Retrieve&dopt=Graphics&list_uids=29089) | UBE2T | Ubiquitin-conjugating enzyme E2T (putative) | 0.099311 | -1.33 |
| [NM_018299](http://www.ncbi.nlm.nih.gov/sites/entrez?db=gene&cmd=Retrieve&dopt=Graphics&list_uids=55284) | UBE2W | Ubiquitin-conjugating enzyme E2W (putative) | 0.785111 | -1.07 |
| [NM_023079](http://www.ncbi.nlm.nih.gov/sites/entrez?db=gene&cmd=Retrieve&dopt=Graphics&list_uids=65264) | UBE2Z | Ubiquitin-conjugating enzyme E2Z | 0.054833 | 1.25 |
| [NM_006048](http://www.ncbi.nlm.nih.gov/sites/entrez?db=gene&cmd=Retrieve&dopt=Graphics&list_uids=10277) | UBE4B | Ubiquitination factor E4B | 0.490662 | -1.12 |
| [NM_174916](http://www.ncbi.nlm.nih.gov/sites/entrez?db=gene&cmd=Retrieve&dopt=Graphics&list_uids=197131) | UBR1 | Ubiquitin protein ligase E3 component n-recognin 1 | 0.750111 | 1.05 |
| [NM_015255](http://www.ncbi.nlm.nih.gov/sites/entrez?db=gene&cmd=Retrieve&dopt=Graphics&list_uids=23304) | UBR2 | Ubiquitin protein ligase E3 component n-recognin 2 | 0.395651 | -1.11 |
| NM_000551 | VHL | Von Hippel-Lindau tumor suppressor | 0.191408 | -1.22 |
| NM_007013 | WWP1 | WW domain containing E3 ubiquitin protein ligase 1 | 0.522541 | -1.06 |
